# Dietary Selenium Intake and Kidney Stones in Old Adults: an Analysis from NHANES 2011 to 2018

**DOI:** 10.1007/s12011-022-03282-8

**Published:** 2022-06-10

**Authors:** Qiao Qi, Yongtao Hu, Yang Chen, Yuexian Xu, Zongyao Hao

**Affiliations:** 1 Department of Urology, the First Affiliated Hospital of Anhui Medical University, Hefei, China; 2 Institute of Urology & Anhui Province Key Laboratory of Genitourinary Diseases, Anhui Medical University, 218th Jixi Road, Hefei, 230022 China

**Keywords:** Dietary selenium, Kidney stones, NHANES

## Abstract

The association between dietary selenium intake and kidney stones remains unclear. The purpose of this study was to explore the correlation between dietary selenium intake and kidney stones in older adults. A total of 6669 adults aged ≥ 60 years who had participated in the National Health and Nutrition Examination Survey (NHANES) during 2011–2018 were enrolled in the current study. The correlation between dietary selenium intake and kidney stones was assessed by the logistic regression analysis. Smooth curve fitting was used to explore the potential non-linear relationship and subgroup analyses were further adopted. After adjustment for multiple confounding factors, the odds ratio (OR) with 95% confidence interval (CI) of kidney stones for per standard deviation increment in dietary selenium intake was 0.92 (0.85, 1.00) overall. Compared with the lowest quartile, the ORs (95% CIs) with increasing quartiles were 0.88 (0.71, 1.08), 0.82 (0.66, 1.02), and 0.79 (0.64, 0.97). In addition, smooth curve fitting and stratified analyses showed that there was a non-linear and stable correlation between dietary selenium intake and the occurrence of kidney stones respectively. For adults aged over 60, dietary selenium intake was inversely correlated with kidney stones, and this relationship remained after adjusting for other confounding variables. Further researches are needed to explore the potential mechanism between dietary selenium intake and kidney stones.

## Introduction

Kidney stone is a relatively prevalent disease worldwide. In America, one in eleven people had ever suffered from kidney stones on average, and in recent years, the incidence rate of kidney stones has been increasing [1]. At the same time, a striking feature of kidney stones is the high rate of recurrence, up to 50% within 5 years [2]. The prevention of kidney stones can not only enhance the quality of life but also reduce the medical economic burden; therefore, it is essential to further identify the risk factors for the disease. Researches have revealed that kidney stones are correlated with many diseases, including obesity [3], diabetes [4], and metabolic syndrome [5]. However, the etiology and pathogenesis are not very clear. At present, it is well known that the occurrence and development of kidney stones are associated with inflammation and oxidative stress, which cause injury to renal tubular epithelial cells [6, 7].

Selenium is an indispensable non-metallic element for the human body with various physiological functions, including anti-oxidation, anti-inflammation, and cleaning up free radicals produced in the body [8, 9]. The inhibitory function of selenium on the occurrence of kidney stones can be found in animal models [10]. Meanwhile, it has been found that compared with astragalus polysaccharides, selenide astragalus polysaccharides have a stronger ability to inhibit stone formation [11], and nano-selenium particles have an obvious negative effect on the aggregation and growth of CaOx crystals [12, 13]. Studies have found that when oxalate crystals are released from damaged thyroid epithelium and colloids, they can cause a series of inflammatory reactions, oxidative stress, and immune reactions (macrophages around oxalate crystals can be observed) [14, 15]. Selenium is a cofactor of many enzymes, and its synthetic selenoproteins (such as glutathione peroxidase and thioredoxin reductase) play an active protective role in Hashimoto disease, thyroiditis, and other diseases [8, 16, 17]. The formation of kidney stones is closely related to the effect of oxalate crystals, so we speculate that selenium intake may be closely related to the formation of kidney stones. However, in large-scale studies, there are few studies focusing on the correlation between selenium intake and kidney stones. Moreover, in elderly individuals, because of their own physiological characteristics and low selenium intake at ordinary times, selenium is easily deficient. Thus, the purpose of this research was to investigate the dose–response relationship between dietary selenium intake and the occurrence of kidney stones in older adults by using NHANES data from 2011 to 2018 while controlling for the effect of confounders on outcomes to reduce error.

## Methods

### Study Population

NHANES is a large-scale survey with representative characteristics of the US population, which aims to collect health-related information on the population of American households. The contents of the project from the NHANES include family interviews and physical examinations. Meanwhile, the survey was conducted in the participants’ homes. At present, NHANES data are continuously collected using stratified multi-stage cluster sampling probability design, and the data are released every 2 years. The NHANES study was authorized by the US ethics committee, and all adults were informed and provided relevant informed consent.

In this analysis, we collected four consecutive NHANES 2-year cycles (2011–2012, 2013–2014, 2015–2016, 2017–2018). There were 46,977 participants in NHANES 2011–2018. The exclusion criteria were described below: (1) missing/unknown kidney stones (*N* = 19,100); (2) missing/unknown dietary selenium intake (*N* = 968); (3) missing/unknown blood selenium (*N* = 6568); (4) age < 60 (*N* = 13,519); and (5) missing/unknown annual family income (*N* = 153). Eventually, 6669 participants in all were included.

### Study Variables and Outcome

The dietary intake data, which were collected in collaboration with the United States Department of Agriculture and the United States Department of Health and Human Services, were used to estimate the amounts and types of drinks and foods consumed in the 24 h prior to the interview (midnight to midnight), and to estimate intakes of nutrients, energy, and other components from those drinks and foods. All participants from NHANES were eligible to participate in two 24-h dietary recall interviews. The first interview was collected in a face-to-face manner, and the second interview was collected by phone inquiry after 3 to 10 days. Therefore, the average dietary selenium intake from the two 24-h recalls was used in this analysis.

In this study, the definition of kidney stones was derived from the participants’ self-reports. The main result of the analysis was the answer to the following question: “Have you ever had kidney stones?” Participants who answered “yes” to this question were thought to have a history of kidney stones.

In addition, we contained numerous covariates that might affect the final result, involving age (≥ 60), gender (male, female), race (Mexican American/other races, non-Hispanic White/Black), body mass index (BMI) (kg/m^2^), moderate recreational activities (yes, no), diabetes (yes, no/unknown), education (< high school, ≥ high school), marital status (married/living with partner, single/widowed/divorced/separated/never married), hypertension (yes, no/unknown), annual family income ($0–$19,999, $20,000–$34,999, ≥ $34,999), smoking (yes, no/unknown), and blood selenium (mcg/L). In addition, the definition of hypertension and diabetes in all participants comes from doctors’ self-diagnosis of hypertension and diabetes. For more information on dietary selenium intake and the process of measuring kidney stones, as well as the process of obtaining other covariates, please visit www.cdc.gov/nchs/nhanes/.

### Statistical Analysis

Continuous variables were represented by the mean and standard deviation, and categorical variables were represented by count and percentage. Chi-square tests and *t* tests were used for categorical variables and continuous variables respectively to describe the baseline characteristics among different dietary selenium intake groups. A multivariate logistic regression model was constructed to estimate the correlation between dietary selenium intake and kidney stones, which was expressed by the ORs with 95% CIs. Model I was adjusted for gender and age. Model II was additionally adjusted for race, marital status, moderate recreational activities, education, hypertension, diabetes, BMI, smoking, blood selenium, and annual family income, with variables from model I. Smooth curve fitting was used to describe the potential non-linear relationship between dietary selenium and kidney stones. In addition, to further evaluate the stability of the results, stratified analyses were conducted according to the following variables: gender, race, BMI, age, marital status, moderate recreational activities, education, hypertension, diabetes, annual family income, and smoking.

In this study, all analysis results were completed by the statistical software packages R3.6.3 (http://www.r-project.org) and Empower Stats (www.empowerstats.net).

## Results

### Participant Characteristics

According to the aforementioned inclusion and exclusion criteria, 6669 people in total were enrolled in the research. The population characteristics of the quartile of the dietary selenium intake are shown in Table 1. It was not difficult to find from Table 1 that the average age of the participants was 69.9 years old, of which 50.13% were men, 32.55% were Mexican American/other races, and 67.45% were non-Hispanic White/Black. Significant differences were identified in age, race, gender, BMI, marital status, moderate recreational activities, education, annual family income, smoking, and blood selenium among dietary selenium intake quartiles, except for hypertension and diabetes.

**Table 1:** Characteristics of the study population by categories of dietary selenium intake in NHANES 2011–2018

Characteristics	Total	Dietary selenium				*P* value
		Q1	Q2	Q3	Q4	
*N*	6669	1666	1665	1450	1888	
Gender, *N* (%)						< 0.001
Male	3343 (50.13)	549 (32.95)	792 (47.57)	829 (57.17)	1173 (62.13)	
Female	3326 (49.87)	1117 (67.05)	873 (52.43)	621 (42.83)	715 (37.87)	
Age (years, mean ± SD)	69.90 ± 6.96	70.45 ± 6.83	70.28 ± 7.01	69.22 ± 6.98	69.60 ± 6.96	< 0.001
BMI (kg/m^2^, mean ± SD)	29.14 ± 6.36	29.37 ± 6.47	29.33 ± 6.53	29.08 ± 5.88	28.81 ± 6.44	0.030
Race, *N* (%)						< 0.001
Mexican American/other races	2171 (32.55)	472 (28.33)	520 (31.23)	442 (30.48)	737 (39.04)	
Non-Hispanic White/Black	4498 (67.45)	1194 (71.67)	1145 (68.77)	1008 (69.52)	1151 (60.96)	
Marital status, *N* (%)						< 0.001
Married/living with partner	3764 (56.44)	805 (48.32)	941 (56.52)	869 (59.93)	1149 (60.86)	
Single/widowed/divorced/separated	2498 (37.64)	861 (51.68)	724 (43.48)	581 (40.07)	739 (39.14)	
Moderate recreational activities, *N* (%)						< 0.001
Yes	2326 (34.88)	568 (34.09)	550 (33.03)	574 (39.59)	634 (33.58)	
No	4343 (65.12)	1098 (65.91)	1115 (66.97)	876 (60.41)	1254 (66.42)	
Education, *N* (%)						< 0.001
< High school	1902 (28.52)	541 (32.47)	467 (28.05)	346 (23.86)	548 (29.03)	
≥ High school	4767 (71.48)	1125 (67.53)	1198 (71.95)	1104 (76.14)	1340 (70.97)	
Hypertension, *N* (%)						0.186
Yes	4119 (61.76)	1053 (63.21)	1033 (62.04)	904 (62.34)	1129 (59.80)	
No/Unknown	2550 (38.24)	613 (36.79)	632 (37.96)	546 (37.66)	759 (40.20)	
Diabetes, *N* (%)						0.366
Yes	1734 (26)	428 (25.69)	444 (26.67)	354 (24.41)	508 (26.91)	
No/Unknown	4935 (74)	1238 (74.31)	1221 (73.33)	1096 (75.59)	1380 (73.09)	
Annual family income, *N* (%)						< 0.001
$0–$19,999	2028 (30.41)	595 (35.71)	482 (28.95)	398 (27.45)	553 (29.29)	
$20,000–$34,999	1353 (20.29)	338 (20.29)	367 (22.04)	288 (19.86)	360 (19.07)	
≥ $34,999	3288 (49.3)	733 (44.00)	816 (49.01)	764 (52.69)	975 (51.64)	
Smoking, *N* (%)						< 0.001
Yes	3306 (49.57)	718 (43.10)	826 (49.61)	758 (52.28)	1004 (53.18)	
No/Unknown	3363 (50.43)	948 (56.90)	839 (50.39)	692 (47.72)	884 (46.82)	
Blood selenium (mcg/L, mean ± SD)	192.62 ± 31.84	190.40 ± 40.61	193.59 ± 27.77	193.12 ± 27.48	193.34 ± 29.33	0.012

*SD*, standard deviation; *BMI*, body mass index

### Dietary Selenium and Kidney Stones

**Fig. 1:**
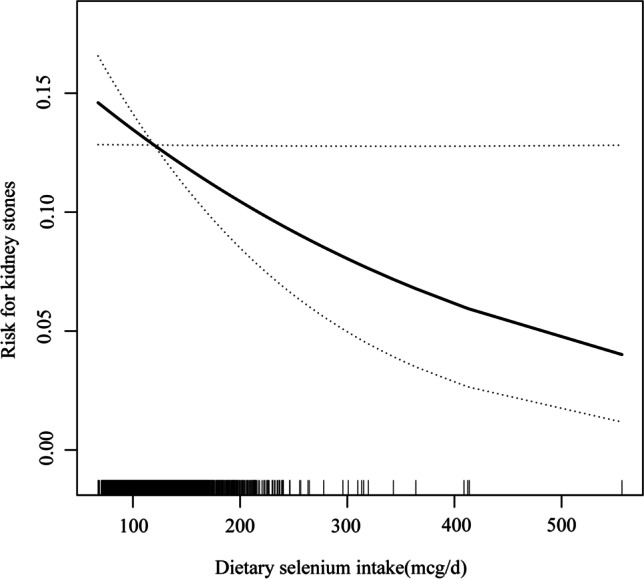
The dose–response relationship between dietary selenium intake and kidney stones. The solid line indicates the estimated risk of kidney stones, and the dotted lines represent a 95% confidence interval from the fit. Adjusted for age, gender, race, marital status, moderate recreational activities, education, hypertension, diabetes, BMI, smoking, blood selenium, and annual family income

In the multivariate logistic regression models, dietary selenium intake was negatively correlated with kidney stones. Table 2 shows the ORs and 95% CIs of the three models. In model II, each SD increase of dietary selenium intake caused an 8% additional risk after adjustment for race, age, BMI, gender, marital status, moderate recreational activities, education, annual family income, smoking, blood selenium, hypertension, and diabetes. Additionally, in the present study, dietary selenium was divided into quartiles, and by comparing with people in the first quartile of dietary selenium intake, the adjusted ORs in quartiles 2–4 were 0.88 (0.71, 1.08), 0.82 (0.66, 1.02), and 0.79 (0.64, 0.97) respectively. Compared with participants in quartile 1, participants in quartiles 2–4 had a prominently lower possibility of kidney stones (OR: 0.83, 95% CI: 0.70, 0.98). Meanwhile, compared with those in quartiles 1–2, participants in quartiles 3–4 also had an obviously lower possibility of kidney stones (OR: 0.86, 95% CI: 0.74, 1.00). Additionally, with full adjustment of all covariates, we used smooth curve fitting (Fig. 1) to depict a negative non-linear correlation of dietary selenium intake with kidney stones, which was consistent with the results described in Table 2.

**Table 2:** Association between dietary selenium intake and kidney stones in different models

Dietary selenium (mcg/d)	Crude model		Model I		Model II	
	OR (95% CI)	*P*	OR (95% CI)	*P*	OR (95% CI)	*P*
Per SD increment	1.00 (0.93, 1.07)	0.944	0.93 (0.85, 1.00)	0.052	0.92 (0.85, 1.00)	0.048
Quartiles						
Q1 (< 101.25)	1.0 (ref.)		1.0 (ref.)		1.0 (ref.)	
Q2 (101.25–116.40)	1.00 (0.82, 1.22)	0.995	0.90 (0.73, 1.10)	0.312	0.88 (0.71, 1.08)	0.216
Q3 (116.40–134.90)	0.97 (0.79, 1.20)	0.774	0.82 (0.66, 1.02)	0.075	0.82 (0.66, 1.02)	0.076
Q4 (> 134.90)	0.98 (0.80, 1.19)	0.822	0.80 (0.65, 0.98)	0.031	0.79 (0.64, 0.97)	0.023
Categories						
Q1 (< 101.25)	1.0 (ref.)		1.0 (ref.)		1.0 (ref.)	
Q2–Q4 (≥ 101.25)	0.98 (0.83, 1.16)	0.838	0.84 (0.71, 1.00)	0.045	0.83 (0.70, 0.98)	0.032
Categories						
Q1–Q2 (< 116.40)	1.0 (ref.)		1.0 (ref.)		1.0 (ref.)	
Q3–Q4 (≥ 116.40)	0.97 (0.84, 1.12)	0.717	0.86 (0.74, 0.99)	0.039	0.86 (0.74, 1.00)	0.047

Model I adjusted for age and gender

Model II adjusted for age, gender, race, marital status, moderate recreational activities, education, hypertension, diabetes, BMI, smoking, blood selenium, and annual family income

### Subgroup Analyses

**Fig. 2:**
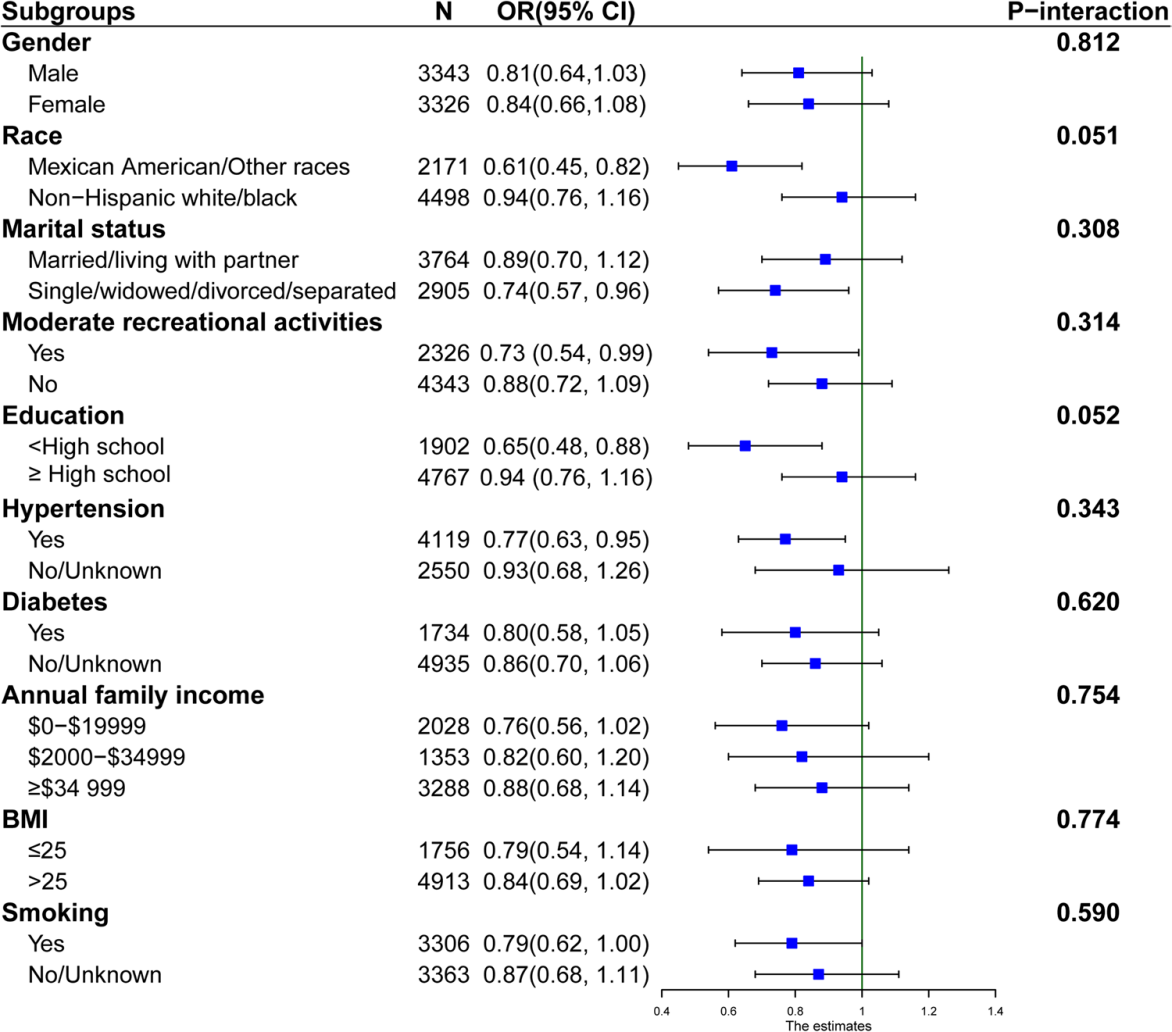
Subgroup analyses for the relationship between dietary selenium intake and kidney stones. The above model adjusted for season of age, gender, race, marital status, moderate recreational activities, education, hypertension, diabetes, BMI, smoking, blood selenium, and annual family income except for the subgroup variable

We further adopted stratified analysis to assess whether the correlation of dietary selenium intake with kidney stones was stable in different subgroups (Fig. 2). After adjustment for gender, race, BMI, age, marital status, moderate recreational activities, education, hypertension, diabetes, annual family income, and smoking, the above variables had no significant interaction apart from stratified variables (all *p* > 0.05).

## Discussion

Kidney stone is a common disease, and the number of people with nephrolithiasis has been on the rise, which brings a heavy medical and health burden [2]. Identifying the factors of stone formation is of great significance for the prevention of kidney stones. In this retrospective study, we first study the association of dietary selenium intake with kidney stones based on the data from the 2011 to 2018 NHANES. After adjustment for gender, age, BMI, race, marital status, physical activity, education, diabetes, hypertension, annual family income, smoking, and blood selenium, multivariate logistic regression showed that dietary selenium intake played a protective role in the occurrence of nephrolithiasis independently. Meanwhile, the dose–response relationship showed that both of them had a strong negative association in all participants after adjusting for confounding variables. In different subgroup analyses, the correlation of both, as we observed, was consistent and stable.

Selenium is an indispensable non-metallic element for the human body and is of great significance in energy metabolism and gene expression [8, 18]. At the same time, selenium has many biological functions, including anti-inflammation, anti-oxidation, immune regulation, anti-aging [9, 19, 20]. Glutathione peroxidase, as the first selenium enzyme discovered, can catalyze the biosynthesis of glutathione and protect cell components such as cell membrane from oxidative damage [17, 21]. In addition, glutathione reductase is another selenium-containing enzyme, which maintains an appropriate level of reduced glutathione to protect cells from the accumulation and damage of hydrogen peroxide [21, 22]. Selenium deficiency can lead to a series of chronic metabolic diseases including atherosclerosis, hyperglycemia, and hyperlipidemia [9]. A prospective cohort study in 589 elderly adults who were followed up for 4 years found that selenium deficiency was related to impaired renal function. Thus, selenium supplementation may give rise to a positive effect on inflammation and oxidative stress, leading to significant improvements in renal function [23]. While, excessive dietary selenium intake will be toxic to the organism. The recommended dietary intake of selenium is 55–79 μg/day for adults. For children aged 4–13, the recommended dietary selenium intake is 30–40 μg/day, while for pregnant or lactating women, the recommended dose is 60 μg/day or more. Meanwhile, the recommended daily dose of selenium also varies by geographic region [21, 24]. Arsenic and cadmium are common environmental pollutants. In daily life, long-term exposure to arsenic and cadmium can damage a variety of tissues and organs including the kidney, which was confirmed in a study using NHANES data sets [25]. Selenium, because of its unique biological function, can protect the kidney from arsenic and cadmium [26, 27]. Sardarabadi et al. found that selenide nanoparticles had an obvious inhibitory effect on the deposition and aggregation of CaOx crystals using transmission electron microscopy and energy-dispersive X-ray analysis [12]. In addition, another study also found that compared with astragalus polysaccharides, selenide astragalus polysaccharides had a stronger ability to inhibit the formation of CaOx crystals and protect cells from COM damage [11]. All mentioned above suggest that selenium has a positive effect on the inhibition of stone formation, which coincides with the results of our study.

Dietary recommendations are related to the type of kidney stones. As for calcium oxalate stones, patients should try to avoid eating foods with high oxalic acid content, and should limit the excessive use of animal protein [28, 29]. For patients with uric acid stones, reducing the intake of high purine foods such as animal viscera may be a better choice. Patients with cystine calculi should try to avoid foods rich in methionine and increase the intake of plant protein and vegetables [30]. Regardless of the type of kidney stones, increasing fluid intake is an important measure to prevent stone formation. In addition, dietary supplement is also closely associated with the occurrence of kidney stones. Due to the very narrow physiological supplement window of selenium, it is easy to lead to selenium deficiency or excess. Therefore, whether to supplement selenium needs reasonable and wise choices [21]. Generally, selenium deficiency is more likely to occur than selenium excess. Selenium deficiency will have adverse effects on the nervous system, cardiovascular system, and immune system. Therefore, it is very necessary to supplement selenium regularly. Studies have shown that the large-scale use of selenium yeast can help reduce the lack of this element caused by the low selenium diet [21, 22]. At the same time, selenium polysaccharide, which has the characteristics of lower toxicity, higher bioavailability, and controllable release, can be used as a potential selenium supplement in the next generation [31]. When the selenium supplement is excessive, especially when taking several supplements at the same time, it is prone to selenium poisoning symptoms. In case of acute selenium poisoning, it is prone to respiratory distress, ataxia, diarrhea, vomiting, and other symptoms. And chronic selenium poisoning is prone to fatigue, depression, garlic smell in breathing, hair loss, and other symptoms [31, 32]. Meanwhile, for people taking drugs, the therapeutic effect of drugs may be weakened. Therefore, dietary supplements should be used in moderation to avoid unnecessary adverse effects.

At present, the specific mechanism between dietary selenium levels and the occurrence of kidney stones is not very clear. Many studies have shown that inflammation and oxidative stress may be the main mechanisms of kidney stone formation [7, 33, 34]. The antioxidant activity of selenium may be one of the mechanisms, which is mainly reflected in the active center of selenoprotein [9, 19], and selenoprotein, as a strong antioxidant protein, plays a beneficial role in many diseases [35–37]. It has been reported that selenium deficiency can induce apoptosis through both endogenous and exogenous pathways, namely the mitochondrial apoptotic pathway induced by oxidative stress and the death receptor pathway induced by inflammatory signals [38]. Moreover, in a rat model of renal injury, we found that selenium could protect the kidney from apoptosis and oxidative stress by inhibiting endoplasmic reticulum stress [39]. This may be part of the mechanism between dietary selenium level and the occurrence of kidney stones. Our research provided potential consequences for the correlation of dietary selenium with kidney stones. Dietary selenium may restrain the occurrence of nephrolithiasis by reducing oxidative stress.

There are some strengths in our research. Primarily, this research contained a considerable number of people who can represent the characteristics of the national population. In addition, the dose–response analysis was used to estimate the relationship between dietary selenium intake and kidney stones. Furthermore, we also conducted a related subgroup analysis which was the advantage of the study. Meanwhile, some limitations should also be noted. First, this is a cross-sectional survey that cannot determine the causal correlation of dietary selenium with the occurrence of kidney stones. Second, although we included as many potential confounding factors as possible, there were still unmeasurable confounding factors, which might impact the accuracy of the conclusion. For example, the information on whether the patients were taking drugs and in what doses, concentrations, times, etc. are unavailable. Finally, due to the limited information retrieved from the NHANES database, the information related to the composition of kidney stones was not included in this research. Therefore, the difference in dietary selenium intake between the classification of renal stones and normal subjects cannot be described and compared.

## Conclusion

Uncovering the relationship between dietary selenium intake and kidney stones provides a fine opportunity to reduce stone disease and health care costs, and ultimately improve health outcomes in older patients. Our study showed that in this nationally representative cross-sectional survey of US residents, dietary selenium intake was inversely associated with kidney stone risk in older adults over 60 years, and this relationship persisted after adjusting for other confounding variables. Further longitudinal studies are needed to determine the potential relationship between dietary selenium intake and kidney stones.

## Data Availability

The datasets generated and analyzed in the present study are available on the website of NHANES datasets 2011–2018 (https://wwwn.cdc.gov/nchs/nhanes).

## References

[1] Scales CD, Smith AC, Hanley JM, Saigal CS, Urologic Diseases in America Project (2012). Prevalence of kidney stones in the United States. Eur Urol.

[2] Khan SR, Pearle MS, Robertson WG, Gambaro G, Canales BK, Doizi S, Traxer O, Tiselius HG (2016). Kidney stones. Nat Rev Dis Primers.

[3] Taylor EN, Stampfer MJ, Curhan GC (2005). Obesity, weight gain, and the risk of kidney stones. JAMA.

[4] Torricelli FC, De S, Gebreselassie S, Li I, Sarkissian C, Monga M (2014). Type-2 diabetes and kidney stones: impact of diabetes medications and glycemic control. Urology.

[5] Kohjimoto Y, Sasaki Y, Iguchi M, Matsumura N, Inagaki T, Hara I (2013). Association of metabolic syndrome traits and severity of kidney stones: results from a nationwide survey on urolithiasis in Japan. Am J Kidney Dis.

[6] Khan SR (2014). Reactive oxygen species, inflammation and calcium oxalate nephrolithiasis. Transl Androl Urol.

[7] Zhu J, Wang Q, Li C, Lu Y, Hu H, Qin B, Xun Y, Zhu Y, Wu Y, Zhang J (2019). Inhibiting inflammation and modulating oxidative stress in oxalate-induced nephrolithiasis with the Nrf2 activator dimethyl fumarate. Free Radic Biol Med.

[8] Rayman MP (2012). Selenium and human health. Lancet.

[9] Wang N, Tan HY, Li S, Xu Y, Guo W, Feng Y (2017). Supplementation of micronutrient selenium in metabolic diseases: its role as an antioxidant. Oxid Med Cell Longev.

[10] Liu Y, Xu H, Zhong W, Shen Q, Zhuang T, Huang K (2015). Organic selenium alleviated the formation of ethylene glycol-induced calcium oxalate renal calculi by improving osteopontin expression and antioxidant capability in dogs. Biol Trace Elem Res.

[11] Huang F, Sun XY, Ouyang JM (2020). Preparation and characterization of selenized astragalus polysaccharide and its inhibitory effect on kidney stones. Mater Sci Eng C Mater Biol Appl.

[12] Sardarabadi H, Mashreghi M, Jamialahmadi K, Matin MM, Darroudi M (2019). Selenium nanoparticle as a bright promising anti-nanobacterial agent. Microb Pathog.

[13] Liang M, Bai Y, Huang L, Zheng W, Liu J (2009). Inhibition of the crystal growth and aggregation of calcium oxalate by elemental selenium nanoparticles. Colloids Surf B Biointerfaces.

[14] Reid JD, Choi CH, Oldroyd NO (1987). Calcium oxalate crystals in the thyroid. Their identification, prevalence, origin, and possible significance. Am J Clin Pathol.

[15] Katoh R, Suzuki K, Hemmi A, Kawaoi A (1993). Nature and significance of calcium oxalate crystals in normal human thyroid gland. A clinicopathological and immunohistochemical study. Virchows Arch A Pathol Anat Histopathol.

[16] Gärtner R, Gasnier BC, Dietrich JW, Krebs B, Angstwurm MW (2002). Selenium supplementation in patients with autoimmune thyroiditis decreases thyroid peroxidase antibodies concentrations. J Clin Endocrinol Metab.

[17] Schomburg L (2011). Selenium, selenoproteins and the thyroid gland: interactions in health and disease. Nat Rev Endocrinol.

[18] Izquierdo A, Casas C, Herrero E (2010). Selenite-induced cell death in Saccharomyces cerevisiae: protective role of glutaredoxins. Microbiology (Reading).

[19] Handy DE, Joseph J, Loscalzo J (2021). Selenium, a micronutrient that modulates cardiovascular health via redox enzymology. Nutrients.

[20] Liu H, Xu H, Huang K (2017). Selenium in the prevention of atherosclerosis and its underlying mechanisms. Metallomics.

[21] Kieliszek M (2019). Selenium-fascinating microelement, properties and sources in food. Molecules.

[22] Kieliszek M, Blazejak S (2013). Selenium: significance, and outlook for supplementation. Nutrition.

[23] Alehagen U, Aaseth J, Alexander J, Brismar K, Larsson A (2020). Selenium and coenzyme Q10 supplementation improves renal function in elderly deficient in selenium: observational results and results from a subgroup analysis of a prospective randomised double-blind placebo-controlled trial. Nutrients.

[24] Kieliszek M, Bano I, Zare H (2022). A comprehensive review on selenium and its effects on human health and distribution in Middle Eastern countries. Biol Trace Elem Res.

[25] Sun Y, Zhou Q, Zheng J (2019). Nephrotoxic metals of cadmium, lead, mercury and arsenic and the odds of kidney stones in adults: an exposure-response analysis of NHANES 2007–2016. Environ Int.

[26] Rahman MM, Uson-Lopez RA, Sikder MT, Tan G, Hosokawa T, Saito T, Kurasaki M (2018). Ameliorative effects of selenium on arsenic-induced cytotoxicity in PC12 cells via modulating autophagy/apoptosis. Chemosphere.

[27] Chen J, He W, Zhu X, Yang S, Yu T, Ma W (2020). Epidemiological study of kidney health in an area with high levels of soil cadmium and selenium: does selenium protect against cadmium-induced kidney injury?. Sci Total Environ.

[28] Shah S, Calle JC (2016). Dietary and medical management of recurrent nephrolithiasis. Cleve Clin J Med.

[29] Coe FL, Evan A, Worcester E (2011). Pathophysiology-based treatment of idiopathic calcium kidney stones. Clin J Am Soc Nephrol.

[30] Chillarón J, Font-Llitjós M, Fort J, Zorzano A, Goldfarb DS, Nunes V, Palacín M (2010). Pathophysiology and treatment of cystinuria. Nat Rev Nephrol.

[31] Constantinescu-Aruxandei D, Frincu RM, Capră L, Oancea F (2018). Selenium analysis and speciation in dietary supplements based on next-generation selenium ingredients. Nutrients.

[32] Lv Q, Liang X, Nong K, Gong Z, Qin T, Qin X, Wang D, Zhu Y (2021). Advances in research on the toxicological effects of selenium. Bull Environ Contam Toxicol.

[33] Qin B, Wang Q, Lu Y, Li C, Hu H, Zhang J, Wang Y, Zhu J, Zhu Y, Xun Y (2018). Losartan ameliorates calcium oxalate-induced elevation of stone-related proteins in renal tubular cells by inhibiting NADPH oxidase and oxidative stress. Oxid Med Cell Longev.

[34] Joshi S, Khan SR (2019). Opportunities for future therapeutic interventions for hyperoxaluria: targeting oxidative stress. Expert Opin Ther Targets.

[35] Reeves MA, Hoffmann PR (2009). The human selenoproteome: recent insights into functions and regulation. Cell Mol Life Sci.

[36] Short SP, Pilat JM, Barrett CW, Reddy VK, Haberman Y, Hendren JR, Marsh BJ, Keating CE, Motley AK, Hill KE (2021). Colonic epithelial-derived selenoprotein P is the source for antioxidant-mediated protection in colitis-associated cancer. Gastroenterology.

[37] Rong Y, Gao J, Kuang T, Chen J, Li JA, Huang Y, Xin H, Fang Y, Han X, Sun LQ (2021). DIAPH3 promotes pancreatic cancer progression by activating selenoprotein TrxR1-mediated antioxidant effects. J Cell Mol Med.

[38] Wang J, Liu Z, He X, Lian S, Liang J, Yu D, Sun D, Wu R (2018). Selenium deficiency induces duodenal villi cell apoptosis via an oxidative stress-induced mitochondrial apoptosis pathway and an inflammatory signaling-induced death receptor pathway. Metallomics.

[39] Zhang Y, Hu B, Wang M, Tong J, Pan J, Wang N, Gong P, Long M (2020). Selenium protects against zearalenone-induced oxidative stress and apoptosis in the mouse kidney by inhibiting endoplasmic reticulum stress. Oxid Med Cell Longev.

